# The association between sodium-glucose cotransporter 2 inhibitor and risk of pancreatic cancer among patients with type 2 diabetes mellitus: A real-world cohort study

**DOI:** 10.1097/MD.0000000000049365

**Published:** 2026-06-19

**Authors:** Yu-Kuan Tu, Yung-Chun Liang, Yu-Jou Wu, Kuo-Chuan Hung, Tsung Yu, Chih-Cheng Lai, Chia-Chen Chen, Jheng-Yan Wu

**Affiliations:** a Department of Internal Medicine, Chi Mei Medical Center, Tainan, Taiwan; b Department of Internal Medicine, National Cheng Kung University Hospital, Tainan, Taiwan; c Division of Cardiology, Department of Internal Medicine, National Taiwan University Hospital, Taipei, Taiwan; d Department of Anesthesiology, Chi Mei Medical Center, Tainan, Taiwan; e Department of Public Health, College of Medicine, National Cheng Kung University, Tainan, Taiwan; f Department of Intensive Care Medicine, Chi Mei Medical Center, Tainan, Taiwan; g School of Medicine, College of Medicine, National Sun Yat-sen University, Kaohsiung, Taiwan; h Division of Endocrinology and Metabolism, Department of Internal Medicine, Chi Mei Hospital, Liouying, Tainan, Taiwan; i Department of Nutrition, Chi Mei Medical Center, Tainan, Taiwan.

**Keywords:** pancreatic cancer, SGLT2i, T2DM

## Abstract

Pancreatic cancer (PC) is one of the deadliest cancers, with limited screening options and a high mortality rate. Sodium-glucose cotransporter 2 inhibitors (SGLT2-i), a novel class of antidiabetic agents for type 2 diabetes mellitus (T2DM), have shown various benefits, but their long-term impact on PC risk is unclear. We conducted a real-world analysis using the TriNetX database, which aggregates de-identified electronic medical records from over 147 million patients across 120 health care organizations in 17 countries. We created 2 cohorts from 7,788,779 patients with newly diagnosed T2DM. One cohort received SGLT2-i, while the other received dipeptidyl peptidase 4 inhibitors (DPP4-i) as an active comparator. Patients were matched for demographics, comorbidities, serum hemoglobin A1c levels, and antidiabetic drug use. The primary outcome was the relative risk (RR) of PC over a 10-year follow-up. SGLT2-i use was associated with a significantly lower risk of PC compared to DPP4-i use (RR = 0.67; 95% CI = 0.613–0.731). Sensitivity analyses supported these findings across different follow-up periods: 3 years (RR = 0.805; 95% CI = 0.727–0.892) and 5 years (RR = 0.748; 95% CI = 0.682–0.82). Long-term SGLT2-i use in patients with T2DM was associated with a reduced risk of PC compared to DPP4-i use. These findings highlight the potential protective role of SGLT2-i against PC. Further studies, including randomized controlled trials, are warranted to clarify the causal relationship.

## 1. Introduction

Pancreatic cancer (PC) is one of the deadliest malignancies worldwide, characterized by its high mortality rate and the lack of effective screening tools.^[1]^ The 5-year survival rate remains as low as 13%,^[2]^ making PC the third leading cause of cancer-related deaths, and it is projected to become the second leading cause by 2040.^[3]^ In addition, the incidence of PC continues to rise at approximately 1% annually, underscoring the need for effective prevention strategies.^[2,4]^

Diabetes mellitus is a well-established risk factor for PC.^[5]^ Several studies have demonstrated that impaired glucose metabolism contributes to PC development and is associated with worse outcomes among affected individuals.^[6–9]^ Consequently, certain oral antidiabetic drugs (OADs) used in the treatment of type 2 diabetes mellitus (T2DM) have been hypothesized to influence PC risk.^[10]^

Sodium-glucose cotransporter 2 inhibitors (SGLT2-i), a newer class of OADs, have shown benefits in glycemic control, body weight reduction, and blood pressure lowering in patients with T2DM.^[11]^ In addition to their cardioprotective and nephroprotective effects,^[12,13]^ Recent preclinical and observational data have suggested potential antitumor properties of SGLT2-i through mechanisms such as inhibition of glucose uptake, reduction of systemic inflammation, and modulation of the tumor microenvironment.^[14–16]^ These findings raise the hypothesis that SGLT2-i may influence the risk of PC in patients with T2DM. However, the evidence remains inconclusive.

Dipeptidyl peptidase 4 inhibitors (DPP4-i), another widely used class of OADs, act by prolonging the action of incretin hormones and improving postprandial glycemic control. Unlike SGLT2-i, DPP4-i has a neutral effect on weight and are not associated with proven cardiovascular or renal benefits.^[17]^ Due to their widespread use and perceived metabolic neutrality, DPP4-i are often used as an active comparator in studies evaluating cancer risk among patients with T2DM.^[18]^

Although prior retrospective and case-control studies have suggested a possible protective effect of SGLT2-i on PC risk,^[16,18]^ their conclusions are limited by small sample sizes and insufficient follow-up durations. To address these limitations, we conducted a real-world, large-scale cohort study using the TriNetX database to investigate the long-term association between SGLT2-i use and the risk of PC among patients with type 2 diabetes.

## 2. Materials and methods

### 2.1. Data source

The data source is from the TriNetX database, which comprises clinical data including demographics, diagnoses (coded using the International Classification of Diseases, Tenth Revision, Clinical Modification [ICD-10-CM]), and medications. TriNetX is a global federated health research network that provides access to electronic medical records from both academic and community hospitals. It aggregates de-identified electronic medical records data from over 147 million patients across 120 health care organizations (HCOs) in 17 countries. The study received ethical approval from the Institutional Review Board of Chi Mei Medical Center (approval number: 11208-E01).

### 2.2. Patient selection and cohorts

This retrospective cohort study is based on an active-comparator new-user design,^[19]^ with DPP4-i serving as the comparator. DPP4-i was selected because both SGLT2-i and DPP4-i are considered newer OADs and are typically prescribed in patients at similar stages of T2DM.^[20–23]^ The study period began in 2013, aligning with the Food and Drug Administration’s approval of SGLT2-i.^[24]^ The index event was the date of the first SGLT2-i or DPP4-i prescription.

We enrolled T2DM patients and classified them based on the administration of OADs. The inclusion criteria were as follows: age ≥ 18 years, diagnosis of T2DM, and a minimum of 2 medical encounters with HCOs from January 1, 2013, to May 31, 2024. We excluded patients who had previously used SGLT2-i or DDP4-i before the index date, those who received a combination of these 2 types of drugs, and those with a history of PC. The SGLT2-i group consisted of patients who received SGLT2-i without combination or prior use of DPP4-i, while the DPP4-i group consisted of patients who received DPP4-i without combination or prior use of SGLT2-i.

### 2.3. Covariates

The covariates and the characteristics of 2 groups within 1 year before the index event including age, gender, races, social determinants of health associated with adverse outcomes, comorbidities, hemoglobin A1c, and other antidiabetic drugs were recorded.^[25]^

### 2.4. Outcomes

The primary outcome was defined as the risk of PC (identified by ICD-10-CM with C25) with a follow-up period up to 10 years. The risk of other 2 time periods, during the 3 years and 5 years, were recorded respectively as sensitivity test. In addition, different types of antidiabetic drugs were analyzed for sensitivity analysis. Finally, we performed subgroup analyses to examine how the results differed according to age, gender, and types of SGLT2-i.

### 2.5. Statistical analysis

The characteristics of the 2 groups are reported using means with standard deviations or counts with proportions. Which compare method was applied for the baseline comparison between groups.

To ensure balanced covariates between 2 groups at baseline, we employed propensity score matching (PSM) using the greedy nearest-neighbor algorithm with a caliper width of 0.1 pooled standard deviations. Adequate matching was indicated by any variable displaying a standardized difference of <0.1 between the groups.^[26]^

After PSM, risk ratio (RR) was calculated to compare the risk of PC between the SGLT2-i group and the DPP4-i group during the 3 follow-up periods. The threshold for significance level was set at 0.05. All the statistical analyses were performed using the TriNetX built-in analysis platform.

### 2.6. Sensitivity analysis

As a sensitivity analysis, we additionally evaluated the association between SGLT-2i and the risk of PC using glucagon-like peptide-1 receptor agonist (GLP-1RA) as an alternative active comparator. Patients initiating SGLT-2is were compared with those initiating GLP-1RAs using the same active-comparator, new-user design, and PSM strategy as in the primary analysis. Covariates included demographics, comorbidities, glycemic control, and concomitant antidiabetic medications assessed within 1 year prior to the index date. Risk estimates were calculated at the network level and reported as pooled effect estimates with 95% confidence intervals, consistent with the analytic framework of the TriNetX platform.

## 3. Results

### 3.1. Demographic characteristics of included patients

**Figure 1.**
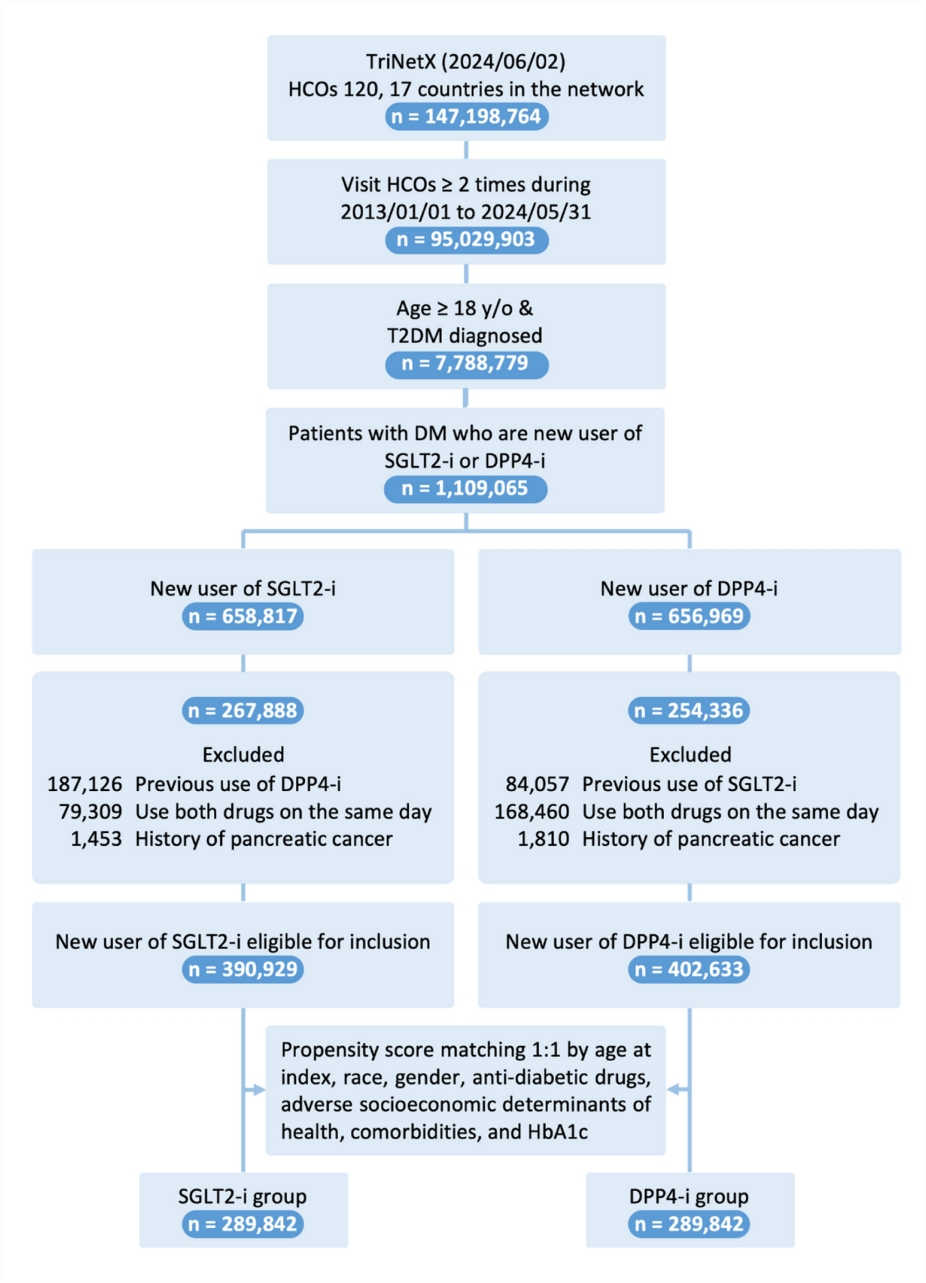
Flowchart of patient selection and cohort construction. DM = diabetes mellitus, DPP4-i = dipeptidyl peptidase 4 inhibitors, HbA1c = hemoglobin A1c, HCO = health care organization, SGLT2-i = sodium-glucose cotransporter 2 inhibitors, T2DM = type 2 diabetes mellitus.

Initial screening of 147,198,764 individuals from 120 HCOs across 17 countries based on the TriNetX platform identified 7,788,779 T2DM patients who visited HCOs at least 2 times from January 1, 2013, to May 31, 2024. Among 1,109,065 new users of SGLT2-i or DPP4-i, the new users of SGLT2-i eligible for inclusion were 390,929 after excluding the previous use of DPP4-i, both drug use, and history of PC. Similarly, 402,633 new users of DPP4-i were eligible for inclusion after excluding those with previous use of SGLT2-i, use of both drugs, and history of PC (Fig. 1).

When compared to patients in the DPP4-i group, those in the SGLT2-i group were younger (mean age: 56.7 ± 12.5 years vs 62.8 ± 13.6 years). In addition, there were notable differences in gender and racial distribution between the 2 groups. Patients in the SGLT2-i group exhibited a lower prevalence of hypertension, dyslipidemia, chronic kidney disease, and cerebrovascular diseases compared to those in the DPP4-i group. Regarding the combined usage of other OADs, excluding SGLT2-i and DPP4-i, biguanides is the most common combined OADs in both groups, followed by sulfonylureas (SU). The SGLT2-i group demonstrated a higher usage rate of glucagon-like peptide-1 (GLP-1) analogues but a lower usage rate of biguanides and SU when compared to the DPP4-i group.

After PSM, our cohort consisted of 289,842 patients who started SGLT2-i treatment and 289,842 patients who started DPP4-i treatment (Table 1). All baseline characteristics were well balanced between both groups after matching (Table 1).

**Table 1:** Baseline characteristics of study population before and after propensity score matching.

Variables	Before matching			After matching		
	SGLT2-i group (n = 390,929)	DPP4-i group (n = 402,633)	Std diff	SGLT2-i group (n = 289,842)	DPP4-i group (n = 289,842)	Std diff
Age at index (yr)						
Mean ± SD	56.7 ± 12.5	62.8 ± 13.6	0.4646	59.2 ± 11.9	59.0 ± 12.8	0.0216
Gender, n (%)						
Female	159,610 (40.8)	198,654 (49.3)	0.1717	129,504 (44.7)	129,656 (44.7)	0.0011
Male	212,326 (54.3)	192,764 (47.9)	0.1290	149,602 (51.6)	149,830 (51.7)	0.0016
Race, n (%)						
White	225,239 (57.6)	202,124 (50.2)	0.1492	160,930 (55.5)	159,192 (54.9)	0.0121
American Indian or Alaska Native	1326 (0.3)	1151 (0.3)	0.0096	927 (0.3)	952 (0.3)	0.0015
Native Hawaiian or Other Pacific Islander	2599 (0.7)	2778 (0.7)	0.0031	2027 (0.7)	2058 (0.7)	0.0013
Black or African American	63,135 (16.2)	55,790 (13.9)	0.0643	45,083 (15.6)	45,704 (15.8)	0.0059
Asian	19,662 (5)	35,296 (8.8)	0.1479	18,132 (6.3)	18,949 (6.5)	0.0115
Other race	13,409 (3.4)	11,280 (2.8)	0.0362	9327 (3.2)	9265 (3.2)	0.0012
Unknown race	65,559 (16.8)	94,214 (23.4)	0.1660	53,416 (18.4)	53,722 (18.5)	0.0027
Social determinants of health associated with adverse outcomes, n (%)						
Housing and economic circumstances	578 (0.1)	508 (0.1)	0.0059	389 (0.1)	399 (0.1)	0.0009
Employment and unemployment	127 (<0.1)	133 (<0.1)	0.0003	106 (<0.1)	100 (<0.1)	0.0011
Education and literacy	68 (<0.1)	56 (<0.1)	0.0028	48 (<0.1)	42 (<0.1)	0.0017
Occupational exposure to risk factors	52 (<0.1)	38 (<0.1)	0.0036	38 (<0.1)	31 (<0.1)	0.0022
Comorbidities, n (%)						
Overweight and obesity	31,595 (8.1)	26,817 (6.7)	0.0544	21,226 (7.3)	20,781 (7.2)	0.0059
Malnutrition	1002 (0.3)	2104 (0.5)	0.0428	892 (0.3)	836 (0.3)	0.0035
Alcohol-related disorders	2384 (0.6)	2322 (0.6)	0.0043	1741 (0.6)	1737 (0.6)	0.0002
Nicotine dependence	12,343 (3.2)	11,871 (2.9)	0.0121	8992 (3.1)	8941 (3.1)	0.0010
Hypertension	96,070 (24.6)	122,720 (30.5)	0.1325	76,441 (26.4)	76,911 (26.5)	0.0037
Hyperlipidemia	81,724 (20.9)	102,350 (25.4)	0.1072	64,189 (22.1)	64,363 (22.2)	0.0014
Chronic lower respiratory diseases	17,212 (4.4)	22,352 (5.6)	0.0528	13,788 (4.8)	13,586 (4.7)	0.0033
Diseases of liver	7336 (1.9)	10,135 (2.5)	0.0437	6027 (2.1)	5781 (2.0)	0.0060
Chronic kidney disease	10,367 (2.7)	27,709 (6.9)	0.1995	9881 (3.4)	9542 (3.3)	0.0065
Cerebrovascular diseases	8161 (2.1)	16,391 (4.1)	0.1150	7375 (2.5)	6790 (2.3)	0.0131
Heart failure	14,766 (3.8)	14,910 (3.7)	0.0039	10,275 (3.5)	9788 (3.4)	0.0092
Atrial fibrillation and flutter	9939 (2.5)	13,560 (3.4)	0.0488	7988 (2.8)	7707 (2.7)	0.0060
Ischemic heart diseases	30,426 (7.8)	35,389 (8.8)	0.0365	22,938 (7.9)	22,724 (7.8)	0.0027
Depression episode	12,313 (3.2)	15,021 (3.7)	0.0319	9746 (3.4)	9612 (3.3)	0.0026
Systemic lupus erythematosus	445 (0.1)	602 (0.2)	0.0098	364 (0.1)	348 (0.1)	0.0016
Acute pancreatitis	1409 (0.4)	1002 (0.2)	0.0202	885 (0.3)	782 (0.3)	0.0066
Lab data						
Serum glucose (mg/dL)	162.5 ± 72.8	163.2 ± 70.2	0.0086	161.9 ± 72.7	166.0 ± 72.0	0.0570
HbA1c (%)	8.0 ± 2.2	7.8 ± 2.0	0.1153	7.9 ± 2.1	7.9 ± 2.1	0.0332
eGFR (mL/min/1.73 m^2^)	74.4.0 ± 21.9	68.3 ± 33.3	0.1985	73.6 ± 27.8	71.4 ± 33.0	0.0718
Antidiabetic drugs						
Biguanides	55,963 (14.3)	76,079 (18.9)	0.1233	45,369 (15.7)	46,612 (16.1)	0.0117
Sulfonylureas	22,783 (5.8)	43,293 (10.8)	0.1793	20,286 (7)	20,693 (7.1)	0.0055
Thiazolidinediones	4666 (1.2)	8523 (2.1)	0.0724	3945 (1.4)	3886 (1.3)	0.0018
Alpha-glucosidase inhibitors	351 (0.1)	1507 (0.4)	0.0592	323 (0.1)	300 (0.1)	0.0024
Glucose-like peptide-1 analogues	11,421 (2.9)	3356 (0.8)	0.1543	4271 (1.5)	3294 (1.1)	0.0297
Insulins and analogues	39,151 (10)	44,225 (11)	0.0316	28,824 (9.9)	28,217 (9.7)	0.0070
SGLT2-i	39,151 (10)	44,225 (11)	0.0316	28,824 (9.9)	28,217 (9.7)	0.0070

Standardized difference (Std diff) < 0.1 is considered a small difference.

DPP4-i = dipeptidyl peptidase 4 inhibitor, eGFR = estimated glomerular filtration rate, HbA1c = hemoglobin A1c, SGLT2-i = sodium-glucose cotransporter 2 inhibitor, Std Diff = standardized difference.

### 3.2. Outcome

For the primary outcome over a 10-year follow-up period, the DPP4-i group exhibited a higher incidence of new PC events compared to the SGLT2-i group (0.43% vs 0.29%). There was also a significantly lower risk of PC in the SGLT2-i group (RR = 0.670; 95% CI = 0.613–0.731; Table 2).

**Table 2:** New events of pancreatic cancer in each time frames and risk ratio comparing SGLT2-i to DPP4-i.

Time frames	Events number/total number		RR (95% CI)
	SGLT2-i	DPP4-i	
Primary analysis			
10 years	829/289,842	1238/289,842	**0.670 (0.613–0.731**)
Sensitivity analysis			
3 years	666/289,842	827/289,842	**0.805 (0.727–0.892**)
5 years	785/289,842	1050/289,842	**0.748 (0.682–0.820**)

Significant results were shown as bold.

CI = confidence interval, DPP4-i = dipeptidyl peptidase 4 inhibitor, RR = risk ratio, SGLT2-i = sodium-glucose cotransporter 2 inhibitor.

**Figure 2.**
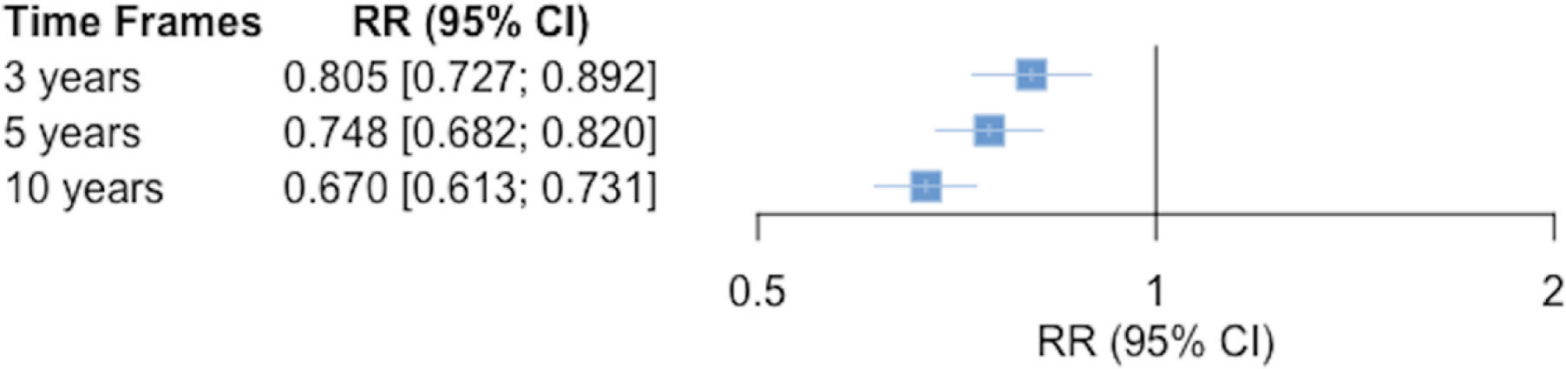
Risk ratio of SGLT2-i compared to DPP4-i for pancreatic cancer. CI = confidence interval, DPP4-i = dipeptidyl peptidase 4 inhibitors, SGLT2-i = sodium-glucose cotransporter 2 inhibitors.

Figure 2 illustrates the temporal trend of RR, showing a decreasing over time. The highest effect size was observed during the 10 years (RR = 0.670; 95% CI = 0.613–0.731), followed by the 5 years (RR = 0.748; 95% CI = 0.682–0.820), and then the 3 years (RR = 0.805; 95% CI = 0.727–0.892).

### 3.3. Subgroup analyses

Subgroup analyses were conducted according to gender, age, types of SGLT2-i and different comparators over the 10-year follow-up period. The results for both genders was consistent with the main outcome (female, RR = 0.7; 95% CI = 0.608–0.805; male, RR = 0.644; 95% CI = 0.571–0.727; Table 3).

**Table 3:** Subgroup analyses and sensitivity analyses of the risk of pancreatic cancer in each time frames.

Variables	Primary analysis – 10 years		Sensitivity analysis – 3 years		Sensitivity analysis – 5 years	
	RR (95% CI)	Interaction *P* value	RR (95% CI)	Interaction *P* value	RR (95% CI)	Interaction *P* value
Gender						
Male	**0.644 (0.571–0.727**)	Ref.	**0.801 (0.696–0.923**)	Ref.	**0.733 (0.644–0.833**)	Ref.
Female	**0.700 (0.608–0.805**)	.3823	**0.805 (0.685–0.947**)	.9639	**0.764 (0.659–0.885**)	.7902
Age						
18–64 y/o	**0.733 (0.604–0.889**)	Ref.	0.860 (0.687–1.077)	Ref.	**0.784 (0.638–0.965**)	Ref.
≥ 65 y/o	**0.645 (0.586–0.710**)	.2670	**0.837 (0.744–0.941**)	.8365	**0.703 (0.635–0.778**)	.3070
Types of SGLT2-i						
Dapagliflozin	**0.547 (0.460–0.651**)	Ref.	**0.720 (0.587–0.884**)	Ref.	**0.610 (0.508–0.732**)	Ref.
Empagliflozin	**0.612 (0.551–0.681**)	.2701	**0.809 (0.718–0.913**)	.3261	**0.738 (0.661–0.825**)	.0707
Canagliflozin	1.273 (1.029–1.576)	**<.0001**	1.148 (0.853–1.546)	**.0261**	1.091 (0.862–1.381)	**.0009**
Comparator						
SU	**0.562 (0.519–0.609**)	Ref.	**0.709 (0.645–0.778**)	Ref.	**0.641 (0.588–0.699**)	Ref.
TZD	**0.644 (0.563–0.737**)	.1008	**0.753 (0.644–0.881**)	.5257	**0.754 (0.654–0.869**)	.0671
GLP-1 RA	1.000 (0.901–1.110)	**<.0001**	1.040 (0.923–1.171)	**<.0001**	1.109 (0.992–1.240)	**<.0001**

Significant results were shown in bold.

CI = confidence interval, DPP4-i = dipeptidyl peptidase 4 inhibitor, GLP-1 RA = glucose-like peptide-1 receptor agonists, Ref. = reference group, RR = risk ratio, SGLT2-i = sodium-glucose cotransporter 2 inhibitor, SU = sulfonylureas, TZD = thiazolidinediones, y/o = years old.

Regarding age, significant differences were observed across all age group (18–64, RR = 0.733; 95% CI = 0.604–0.889; older than 65, RR = 0.645; 95% CI = 0.586–0.71; Table 3).

Both dapagliflozin and empagliflozin use were associated with the significant reduction in the risk of PC (dapagliflozin, RR = 0.547; 95% CI = 0.460–0.651; empagliflozin, RR = 0.612; 95% CI = 0.551–0.681). In contrast, canagliflozin might be associated with a higher risk of PC (RR = 1.273; 95% CI = 1.029–1.576; Table 3).

Lastly, there was a significant interaction effect between types of SGLT2-i and OADS (Table 3). Canagliflozin significantly differed from dapagliflozin across all time frames, and GLP-1 significantly differed from SU across all time frames (Table 3).

### 3.4. Sensitivity analysis

Sensitivity analyses were conducted to evaluate the effects of SGLT2-i use on the risk of PC. As shown in Table 2, significant reductions in the RR of PC were observed in both the 3-year follow-up period (RR = 0.805; 95% CI = 0.727–0.892) and the 5-year follow-up period (RR = 0.748; 95% CI = 0.682–0.82), consistent with the main findings at the 10-year follow-up period.

In the subgroup analyses of the sensitivity results, robust findings were observed across both gender and age groups, with the exception of the 18 to 64 age group during the 3 years follow-up period. Significant results were also noted across different types of SGLT2-i and OADS, except for canagliflozin, which did not show significant effects across the 3 time frames (Table 3).

**Figure 3.**
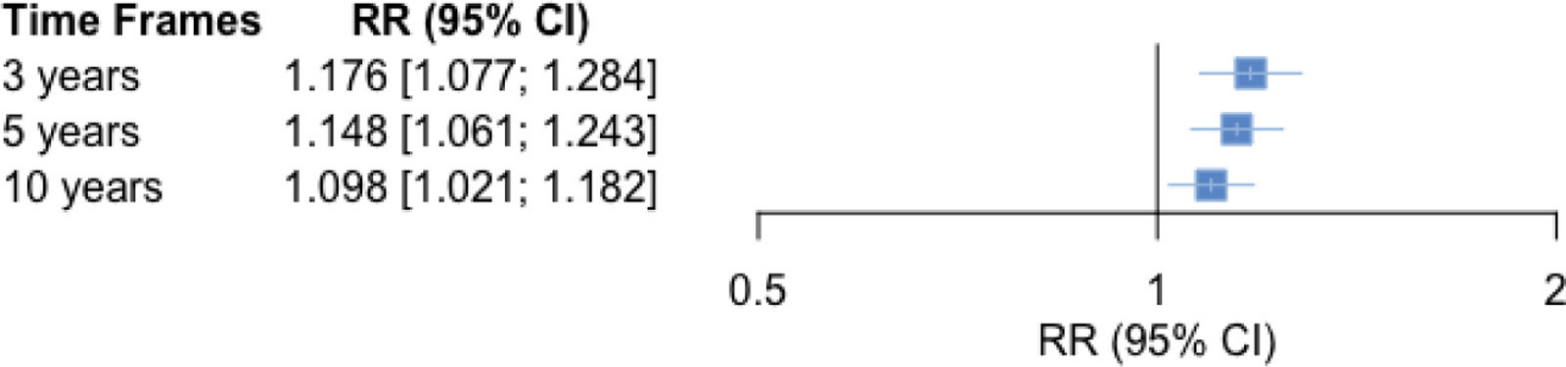
Risk ratio of SGLT2-i compared to GLP-1RA for pancreatic cancer. GLP-1RA = glucagon-like peptide-1 receptor agonist, SGLT2-i = sodium-glucose cotransporter 2 inhibitors.

Over a 10-year follow-up period, the incidence of PC was 1445 events among 358,745 patients in the SGLT-2i group and 1430 events among 358,745 patients in the GLP-1RA group, corresponding to a higher risk in the SGLT-2i group (RR = 1.098; 95% CI = 1.021–1.182; Fig. 3).

Consistent findings were observed in shorter follow-up periods. At 3 years, the risk of PC was higher among SGLT-2i users compared with GLP-1RA users (RR = 1.176; 95% CI = 1.077–1.284), and similar results were noted at 5 years (RR = 1.148; 95% CI = 1.061–1.243; Table 4).

**Table 4:** New events of pancreatic cancer in each time frames and risk ratio comparing SGLT-2i to GLP-1RA.

Time frames	Events number/total number		RR (95% CI)
	SGLT-2i	GLP-1RA	
Primary analysis			
10 years	1445/358,745	1430/358,745	**1.098 (1.021–1.182**)
Sensitivity analysis			
3 years	1057/358,745	933/358,745	**1.176 (1.077–1.284**)
5 years	1281/358,745	1178/358,745	**1.148 (1.061–1.243**)

Significant results were shown as bold.

CI = confidence interval, GLP-1RA = glucagon-like peptide-1 receptor agonist, RR = risk ratio, SGLT-2i = sodium-glucose cotransporter 2 inhibitor.

## 4. Discussion

In this real-world study involving 579,684 patients, we compared the risk of PC between newly SGLT2-i and DPP4-i use in T2DM patients. The result of present study demonstrated that compared to DDP4-i use, SGLT2-i use was associated with a significantly reduced risk for PC over a 10-year follow-up period (RR = 0.67; 95% CI = 0.613–0.731). Sensitivity analyses conducted at multiple time intervals confirmed the inverse association between SGLT2 inhibitor use and the risk of PC, with consistent findings observed at 3 years (RR = 0.805; 95% CI = 0.727–0.892) and 5 years (RR = 0.748; 95% CI = 0.682–0.820) of follow-up. These results suggest that the potential protective effect of SGLT2 inhibitors against PC may be sustained over time.

SGLT2-i has been reported to have antiproliferative effects due to their blockage of glucose reuptake in cells and suppressing glycolysis via the PI3K/AKT/mTOR pathway.^[27,28]^ Besides, the effect of SGLT2-i on DM control may destroy the adequate microenvironment for cancer cell proliferation and survival by reducing proinflammatory conditions caused by DM-induced cytokines.^[29–31]^ These inflammatory process was also reported to be possible cause of PC.^[32]^ The other study also suggest that DM may induced cellular processes by dysregulated microRNAs which involved in PC development and progression.^[33]^ Given the possible pathophysiology above, SGLT2-i use in T2DM patient may have protective effect on PC.^[16,18,34,35]^

Our main result is consistent with the previous nested case-control study and the other retrospective cohort study, which suggest that SGLT2-i use was associated with a lower risk of PC in T2DM patients.^[16,18]^ The retrospective cohort study based on the Taiwan National Health Insurance database, which tracing SGLT‐2i cohort comprised 150,061 patients and DPP‐4i cohort comprised 234,027 patients for 6 years, demonstrated a significant lower risk of developing PC in SGLT-2i cohort while comparing DPP-4i (adjusted hazard ratios = 0.90; 95% CI = 0.87–0.93).^[18]^ The nested case-control study conducted using the Japan Medical Data Center administrative claims database including 363 patients using SGLT2-i and 7043 controls separately from January 2005 to June 2020, revealed that cumulative administration of SGLT2-i for more than 180 days was significantly inversely associated with the development of PC (adjusted OR = 0.58; 95% CI = 0.31–0.99).^[16]^ Both studies were based on national medical data and consisted mainly of Asian populations. In contrast, our study was based on TriNetX database included a large and diverse population, rendering its findings more applicable to real-world scenarios. In addition, our follow-up period extended up to 10 years, providing long-term risk of PC assessment, minimizing random errors, and enhancing the statistical power and reliability of the research.

While randomized controlled trials (RCTs) have not demonstrated a statistically significant difference in PC risk, they were not designed to assess cancer outcomes. Two meta-analyses of RCTs showed no significant difference in PC incidence between SGLT2-i users and those receiving other OADs or placebo.^[34,35]^ However, these results should be interpreted cautiously due to methodological limitations: most trials were short in duration (<3 years), lacked cancer-specific endpoints, and involved highly selected populations that may not reflect real-world clinical settings.

An important exploratory finding from our study was the suggestion of a possible increased risk of PC associated with canagliflozin. This contrasts with preclinical data suggesting that canagliflozin suppresses tumor growth through glycolysis inhibition,^[36]^ yet other studies have raised concerns that canagliflozin may promote cancer cell survival under certain conditions.^[37]^ The CANVAS trials also highlighted an increased incidence of bladder cancer with canagliflozin, raising concerns about its safety profile. While the exact mechanisms remain unclear, canagliflozin’s unique adverse effects, such as increased amputation risk, may contribute to systemic inflammation, a known cancer risk factor.^[38]^ These observations warrant cautious use of canagliflozin in populations at high cancer risk.

Our study has several strengths. To our knowledge, this is the first real-world cohort study using the TriNetX platform to assess the association between SGLT2-i and PC risk in patients with T2DM. We encompass a large and diverse population, rendering its findings more applicable to real-life scenarios. This critical aspect is particularly relevant in the context of patients with and lead to better external validity and generalizability, implying stronger extrapolation capabilities. We defined populations with DPP4-i use as control group because DPP4-i served as an OAD which had been widely used as oral glucose-lowering agents with a well-established neutral profile regarding body weight, cardiovascular risk, and renal outcomes.^[39–41]^ These characteristics make DPP-4 inhibitors a clinically appropriate comparator in observational studies evaluating the long-term safety of antidiabetic therapies. In addition, the 10-year follow-up allowed us to examine temporal trends in risk, providing clinicians with insight into time-related benefit following SGLT2-i initiation.

This study has several limitations. First, the diagnosis of PC was based on ICD-10 codes without confirmation of clinical staging or histopathologic findings, which may have led to misclassification and underestimated disease severity. Second, drug exposure was determined from filled prescriptions, without confirmation of actual adherence. The absence of data on drug dosage, treatment duration, and cumulative exposure may have introduced exposure misclassification, as pharmacokinetics and pharmacodynamics may influence cancer risk. Third, the number of events in certain subgroup analyses was low, resulting in wide confidence intervals and reduced statistical precision. In addition, unmeasured confounders – such as smoking status, alcohol use, genetic predisposition, and dietary factors – could not be fully accounted for, and residual confounding remains a key limitation inherent to observational studies.^[42,43]^ Finally, given the epidemiologic nature of the study, the observed association between SGLT2 inhibitor use and PC risk should not be interpreted as causal. Further prospective cohort studies and RCTs are warranted to confirm our findings and elucidate the potential mechanisms involved.

## 5. Conclusion

Long-term use of SGLT2-i in patients with T2DM was associated with a reduced risk of PC compared to DPP4-i use. These findings suggest a potential protective effect of SGLT2-i against PC. However, it is noteworthy that canagliflozin may be associated with an increased risk. Further investigation, including RCTs, is warranted to elucidate the causal relationship.

## Author contributions

**Visualization:** Yu-Kuan Tu.

**Writing – original draft:** Yu-Kuan Tu.

**Writing – review & editing:** Yung-Chun Liang.

**Conceptualization:** Yu-Jou Wu, Jheng-Yan Wu.

**Formal analysis:** Kuo-Chuan Hung.

**Software:** Tsung Yu.

**Validation:** Chih-Cheng Lai.

**Methodology:** Chia-Chen Chen.
